# Interactive visual exploration of surgical process data

**DOI:** 10.1007/s11548-022-02758-1

**Published:** 2022-10-21

**Authors:** Benedikt Mayer, Monique Meuschke, Jimmy Chen, Beat P. Müller-Stich, Martin Wagner, Bernhard Preim, Sandy Engelhardt

**Affiliations:** 1 Department of Simulation and Graphics, University of Magdeburg, Magdeburg, Germany; 2 Department of Cardiac Surgery, Group Artificial Intelligence in Cardiovascular Medicine, University of Heidelberg, Heidelberg, Germany; 3 Department of General, Visceral and Transplantation Surgery, Heidelberg University Hospital, Heidelberg, Germany

**Keywords:** Surgical workflow, Visualization, Surgical data science

## Abstract

**Purpose:**

Integrated operating rooms provide rich sources of temporal information about surgical procedures, which has led to the emergence of *surgical data science*. However, little emphasis has been put on interactive visualization of such temporal datasets to gain further insights. Our goal is to put heterogeneous data sequences in relation to better understand the workflows of individual procedures as well as selected subsets, e.g., with respect to different surgical phase distributions and surgical instrument usage patterns.

**Methods:**

We developed a reusable web-based application design to analyze data derived from surgical procedure recordings. It consists of aggregated, synchronized visualizations for the original temporal data as well as for derived information, and includes tailored interaction techniques for selection and filtering. To enable reproducibility, we evaluated it across four types of surgeries from two openly available datasets (HeiCo and Cholec80). User evaluation has been conducted with twelve students and practitioners with surgical and technical background.

**Results:**

The evaluation showed that the application has the complexity of an expert tool (System Usability Score of 57.73) but allowed the participants to solve various analysis tasks correctly (78.8% on average) and to come up with novel hypotheses regarding the data.

**Conclusion:**

The novel application supports postoperative expert-driven analysis, improving the understanding of surgical workflows and the underlying datasets. It facilitates analysis across multiple synchronized views representing information from different data sources and, thereby, advances the field of surgical data science.

**Supplementary Information:**

The online version contains supplementary material available at 10.1007/s11548-022-02758-1.

## Introduction

With the success of machine learning, surgery is enriched by data-driven techniques. Therefore, the research field of *surgical data science* [1] has emerged. Congruent is the ongoing technological development of operating room suites (OR). Such suites have undergone a tremendous change, which resulted in digital and integrated OR. Previously, mainly isolated passive medical devices were in use, and device information and processes within the operating theaters were barely considered as an information source. Nowadays, integrated OR evolved to functionally connect the OR environment, which means that patient monitoring, surgical lights, room lights, imaging devices, and further specialized equipment can communicate with each other and their states can be captured in data streams [2].

So far, the available data have been used to solve machine learning tasks such as estimation of tool presence or surgical phase prediction [3]. Beyond that, a more detailed preoperative planning and postoperative representation of the surgery is possible based on such data [4]. However, it is under-investigated how to deal with the increasing and diverse data load to enable expert-driven analysis of the data sets. Corresponding systems need to include adequate querying mechanisms and visual exploration techniques. Accordingly, we provide a design and openly available implementation to support the following scenario.

## Related work

### Surgical workflows and process models

Surgical procedures of the same type usually follow a certain workflow, consisting of different events with a certain temporal extent. To enable consistent naming of such events, corresponding terminology is commonly defined. This terminology may describe the process on different levels of granularity, e.g., it is typical to refer to high-level events during surgery as *phases* [5]. Individual phase lengths and phase occurrence patterns vary between surgeries and are, therefore, subject to further analysis. Additional terminology has developed for more low-level events than phases, like *steps* and *actions* [6, 7], introducing hierarchical relations between the events. Neumuth et al. refer to the visualization of surgical workflows as one of multiple levels of abstraction of surgical procedures [8] and proposed a timeline-based and logic-oriented visualization of such event-based information [4]. Other techniques to represent surgical workflow models include hidden Markov model graphs and line charts [9, 10]. Additionally, time-oriented data like phase annotations are often represented as sequences of bars [8, 10]. However, these techniques do not scale well if larger numbers of procedures or events should be displayed. Nor do they provide solutions for performing combined analysis of data coming from different sources, like phase and instrument annotations, or device measurements. We aim to allow such investigations in our work. In the notion of Lalys and Jannin [5], we facilitate “Analysis” in the form of “Display” to “Explore qualitatively and illustrate results,” and support the “Aggregation - Registration” of surgical procedure data.

The formalization of surgical procedures as sequential lists of events is referred to as *surgical process modeling* [5]. Neumuth et al. distinguish between *individual surgical process models* describing a single procedure and *generic surgical process models* [11]. The latter are referred to as “mean” interventional models computed from individual process models. Such a generic model can be useful in statistical assessment, visualization, and quantification of deviation from the mean procedure. However, the work does not provide any visual tools in this direction.

Focusing more on automated analysis, techniques from *sequence analysis* can be used to compare and evaluate surgical procedures [12]. In general, visualizations do not play a central role in surgical process mining, yet. Accordingly, Rojas et al. call for a better “visualization of the process models and the results obtained” [13].

### Related datasets

There exist datasets related to the scenario from Section 1, covering information like event annotations, instrument presence, or device measurements. (Below, we write the different event types italicized.)

The dataset *SARAS* consists of four procedure recordings with approximately 27 thousand frames in total. In them, 21 different *action classes* were annotated [14]. In contrast, *MISAW* comprises video data, kinematic data, and workflow annotations [6]. The annotations consist of hierarchically ordered events: *phases* > *steps* > *activities*. In total, 27 sequences were recorded from laparoscopic surgeries. Another dataset, provided by Charriére et al., contains 50 videos of cataract surgeries [15], where each frame is annotated with one out of 19 different surgical *steps*. Moreover, Wagner et al. collected 33 videos from laparoscopic surgeries [7], including annotations of seven *phases*, four surgical *actions*, presence of 21 instruments, and surgical skill classifications.

**Fig. 1:**
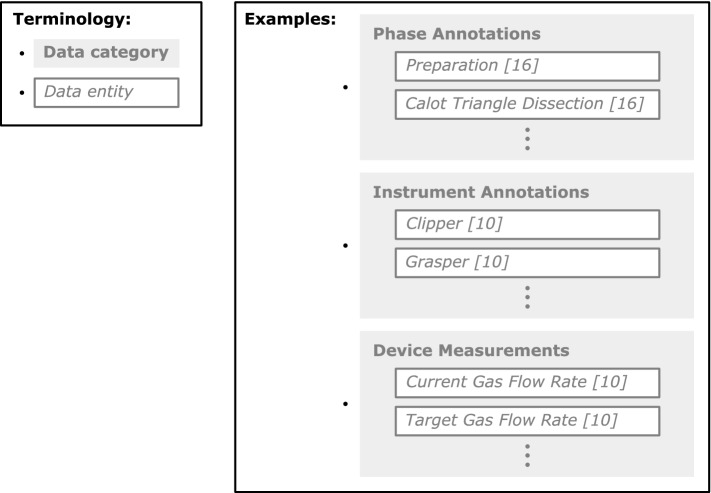
The figure gives examples (taken from *Cholec80* [16] and *HeiCo* [10]) for the terms *data category* and *data entity*

We used the following two datasets. *HeiCo* comprises 30 videos from three types of laparoscopic surgeries, namely proctocolectomy surgeries as well as rectal and sigmoid resections [10]. It also includes annotations of the surgical *phases*, the presence of instruments, and measurements from medical devices. The *phases*, instruments, and devices are the same across all three surgery types. In addition, we used *Cholec80*, which contains 80 videos of laparoscopic surgeries, including *phase* and instrument annotations [16].

We selected *HeiCo* and *Cholec80* as they are open source and cover a variety of data sources and types. Hence, our solution can be applied to other datasets of the same variety. Moreover, *Cholec80* with its 80 procedures allows us to test whether our solution can also scale to larger datasets. Note that we excluded hierarchical data from our investigations to narrow the scope.

## Analysis goals

We identified the main analysis goals for the scenario described in Section 1, excluding tasks focused on the automatic inference of knowledge from the data.

### Terminology

To introduce the goals, we use the following terminology (see Fig. 1 for examples).

- **Data category:** The sum of all information coming from one time-dependent source is considered a *data category*. Aside from the examples given in Fig. 1, this also includes events like surgical actions, covered in other data sets [6, 7].
- **Data entity:** Individual recording entities for the different data categories are referred to as *data entities*.

### Main goals

We identified the following three main analysis goals:

**Synchronize information** ($G_S$). The first goal is to synchronize the information from different procedures and from different data categories. For this, the time-dependent information from the different data entities needs to be temporally aligned within each procedure. It also requires options to align the information from different procedures. This presents a particular challenge since procedures can differ in their execution, raising the question of which point in one procedure corresponds to which point in another procedure.

**Compare information** ($G_C$). The second goal covers the comparison between different procedures for the same data entity, but also the comparison across data entities. In the first case, multiple approaches are desirable: New insights can be gained by comparing procedures *one vs. one*, *one vs. many*, and *many vs. many*. To facilitate such comparisons, means of aggregation, filtering, and selection need to be provided. Moreover, for comparisons across procedures or data entities, the data needs to be synchronized first ($G_S$).

**Understand the workflow** ($G_W$). The third goal is to better understand the surgical workflows in practice. This should be possible at the level of individual procedures as well as groups of procedures. An improved understanding of the workflows can help experts to form new hypotheses from the data, e.g., by learning how the average procedures are executed and comparing them to exceptional ones, or understanding the usage patterns of the surgical instruments. Such investigations rely on means for synchronization ($G_S$), aggregation, and comparison ($G_C$).

## Design of the Application

We created a design to meet the goals identified in Section 3. It consists of three classes of visualizations and tailored interaction options. With the introduction of the classes below, we state which characteristics a dataset must have to make the visualization applicable. For the related examples, we use the dataset *HeiCo* [10] since it contains diverse qualitative and quantitative information. We also implemented the proposed design as a web-based application at https://isgwww.cs.ovgu.de/~benedikt/endovis_workflow/ and created an explanatory video of it.

**Fig. 2:**
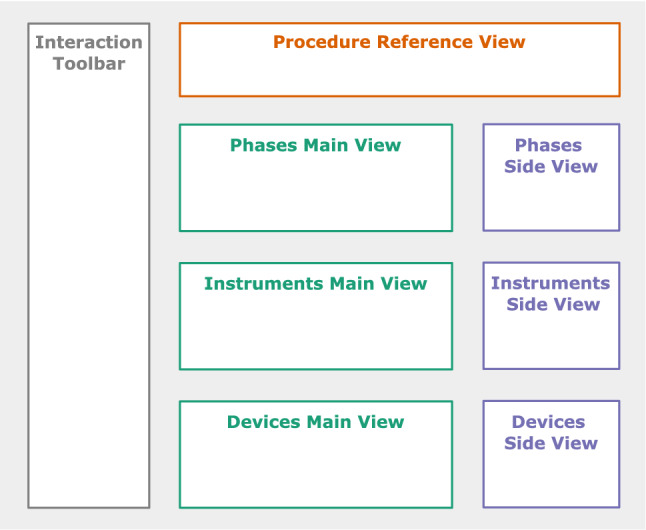
A schematic representation of the main components of the design

### Reference view

The design contains a reference view at the very top (see Fig. 2) to allow the users to refer to the individual procedures at any time.

**Fig. 3:**
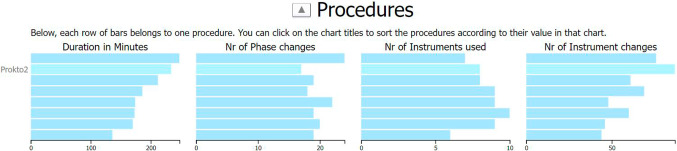
The procedure reference view provides an overview of the individual procedures, with the highlighted bars belonging to a single procedure, here with the label “Prokto2”

*Procedure reference view.* Each procedure is represented by a row of bars (see Fig. 3). The length of each bar represents a time-independent value for the corresponding procedure. Here, we only include information calculated across each procedure, but other information could be presented in this view, too, like surgical skill classifications [7] or the similarity between procedures [12].

### Main views

**Fig. 4:**
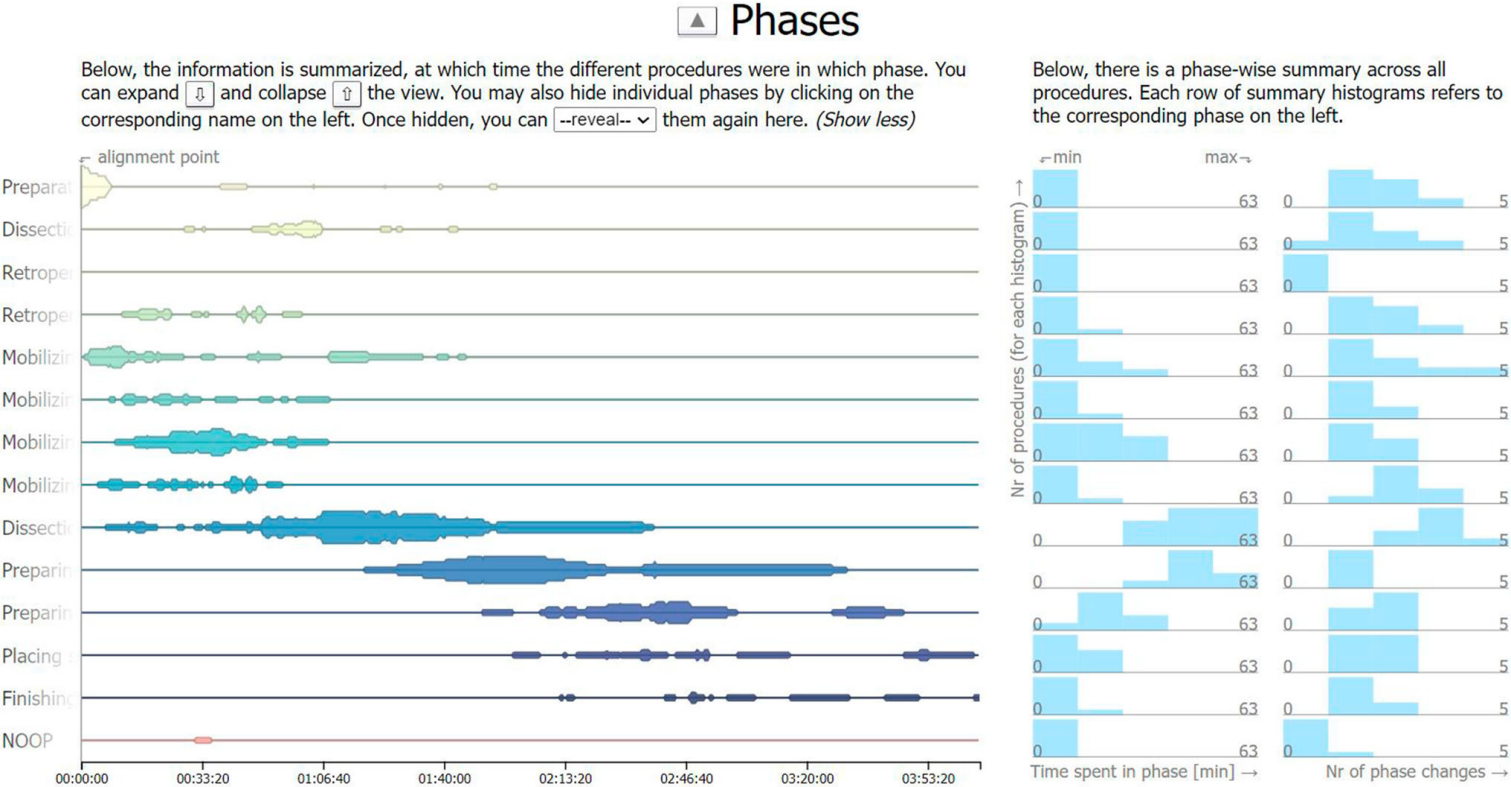
The phases view consists of the time-dependent main view (left) and the derived side view (right)

We propose one column in the center consisting of one main view for each of the time-dependent data categories (see Fig. 2). For each category, we provide a view with the temporal axis in the horizontal direction and the different data entities in the vertical direction. The main views are positioned such that their temporal axes are aligned to allow a synchronized inspection ($G_S$) and comparison ($G_C$) of the data categories and entities. On each of the temporal axes, the information from the individual procedures is aligned at the *alignment point*, which is represented as a vertical line and defaults to the beginning of the first phase (see Fig. 4). We now present the views we propose for the different data categories.

*Phases main view.* The topmost view is used for the data category that divides the procedures into the most coarse events, i.e., the phase annotations in case of *HeiCo* [10]. The phase annotation data is representative of data categories whose data entities are conceptually ordered while the entities contain binary information for each procedure. For each entity, i.e., phase, we map the information of how many procedures were in the given phase throughout the surgery to a horizontally connected set of areas (see Fig. 4, left). The higher the area, the more procedures were in the phase at that time. Thus, the information from different procedures is aggregated and can be compared ($G_C$).

**Fig. 5:**
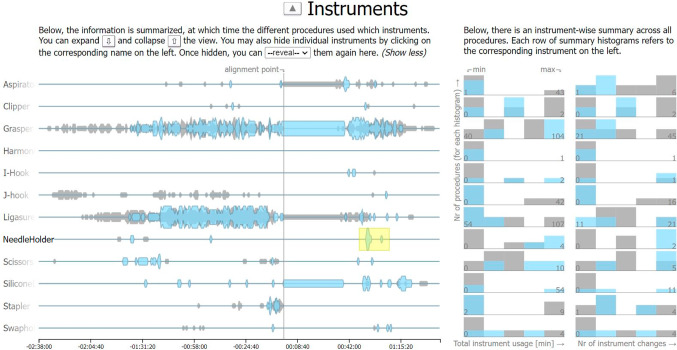
Based on a highlight constraint (yellow rectangle), a subset of procedures is highlighted (blue) to be compared to the remaining procedures (gray). The alignment point is placed at around 60% of the temporal axis in the main view (left)

To represent the conceptual order of the phases, we color the areas based on a sequential colorblind-safe scale. The “NOOP” phase, which represents an exceptional phase that does not normally occur during the procedure, was given a bright red color. If two subsets of procedures should be compared, the areas corresponding to one set are kept colored and the fill color of the other set of areas is changed to gray (e.g., see Fig. 5). More details on how such subsets can be selected are given in “Interaction Techniques” Additionally, the abbreviated phase name labels on the left are fully revealed when hovering over them.

*Instruments main view.* The second view is used for the instrument annotations. The data entities for the instruments do not have an inherent order but, again, contain binary information for each procedure. Hence, the view is constructed in the same way as the phases view, except that only a single main fill color is used for the areas (see Fig. 5).

**Fig. 6:**
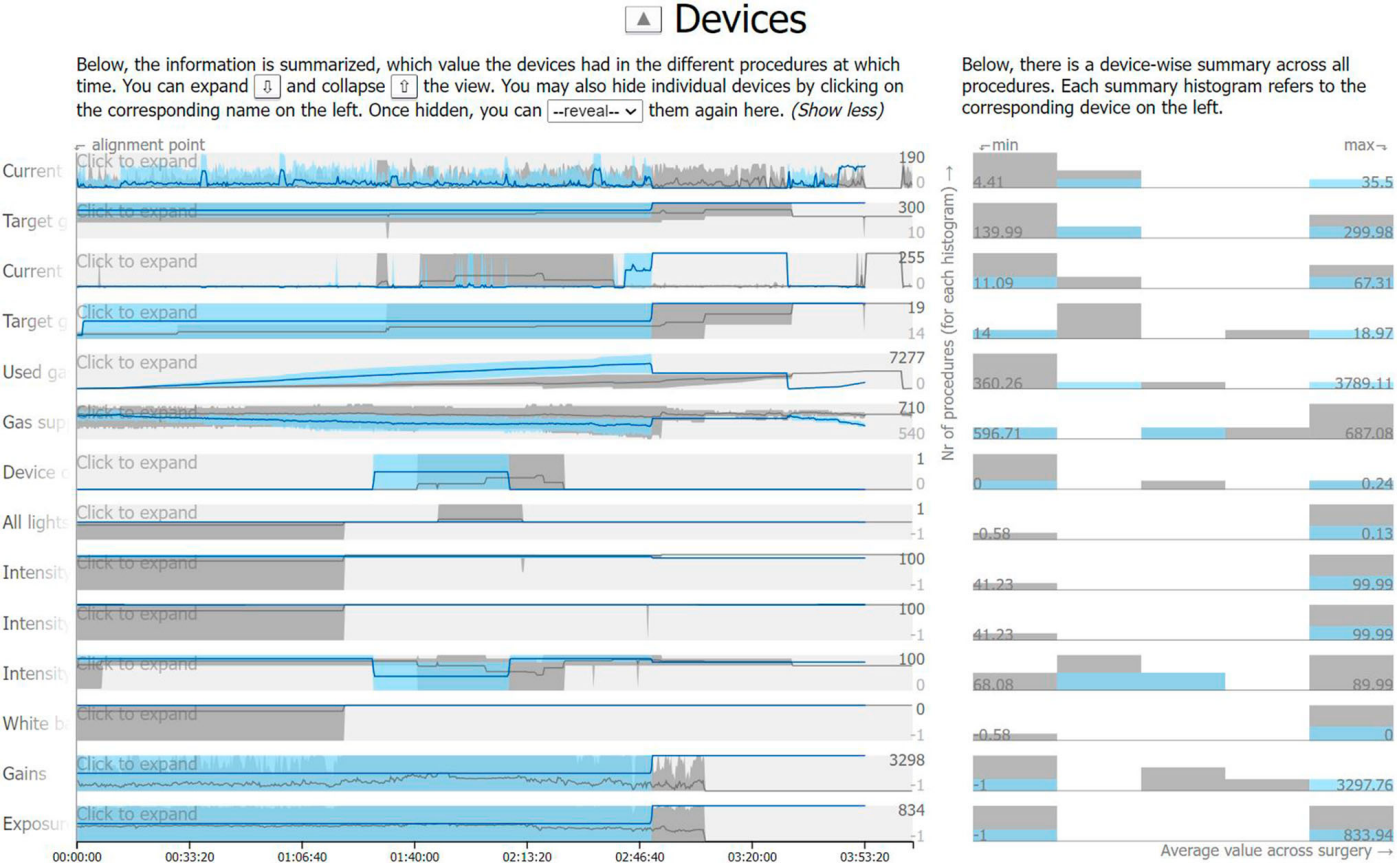
A subset of procedures is highlighted (blue) in the devices view to be compared to the remaining procedures (gray)

*Devices main view.* The data entities in this view, the devices, are again without inherent order. However, the entities contain continuous instead of binary information. To represent the information from multiple procedures visually, two forms of aggregation need to be performed:

The first form refers to each procedure individually. Device values are often measured at a higher frequency (e.g., 25 per second) than there are pixels in a single row of a common computer screen. Hence, multiple measured values need to be represented in the width of a single pixel. We do so by extracting the minimum (min), maximum (max), and mean value of all values corresponding to a single pixel. This way, we can depict a single procedure through the development of its min, max, and mean values over time. We map the mean value to a dark blue line and draw the line over a bright blue area of which the upper edge represents the max values and the lower edge the min values (compare Fig. 6). We selected the min/max solution over more precise representations of the distribution since it is the least complex to understand.

The second form of aggregation is performed across all procedures, not only a single one, to represent their average developments over time. To do so, we calculate the min, max, and mean value for all frames corresponding to a single pixel across *all* procedures. The result can be represented like for a single procedure (see Fig. 6).

**Fig. 7:**
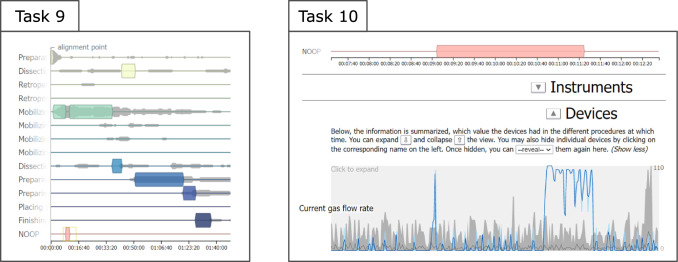
The phases main view is used to solve tasks 9 and 10 from the evaluation, in task 10 in combination with the devices main view

To improve the readability, individual device rows can be expanded for a closer inspection (see Fig. 7). Moreover, if two subsets of procedures should be compared, the visual representations of one of the sets are kept colored while the others are colored in gray.

### Side views

In addition to the main views, we propose a column of side views containing one view for each data category (see Fig. 2) to represent the time-*in*dependent information for each of the data entities. One goal was to facilitate the identification of procedures of interest, e.g., those which stayed particularly long in a certain phase, did not use a certain instrument, or recorded extreme values for a certain device ($G_C$). Additionally, the views should support the analysis of the relations between different data entities ($G_W$). Accordingly, we selected a set of histograms showing summarized information, but other data could be displayed there, too. Each histogram shows how the procedures are distributed regarding a certain value derived for the corresponding data entity. For our selected datasets, we used the following derived information.

*Phases side view.* The histograms depict how much time the different procedures have spent in each phase, and how often the procedures stepped into each of the phases (see Fig. 4, right).

*Instruments side view.* The histograms show for how long each instrument was used in the different procedures, and how often the instruments were changed in them (see Fig. 5, right).

*Devices side view.* We include one histogram for the devices, depicting the average value the procedures had across the surgery (see Fig. 6, right).

### Interaction Techniques

We included various interaction techniques to adjust the views. Most of them can be reached via the interaction toolbar (see Fig. 2).

**Alignment.** The users can adjust the *alignment point* at which the time-dependent information should be aligned. The point can be set to either the first or the last occurrence of any of the phases. Accordingly, all procedure recordings in the main views are shifted along the time axis to be aligned at the point where the corresponding phase first/last occurred in them (e.g., see Fig. 5). This way, the information of all data entities from all data categories can be synchronized ($G_S$) and compared ($G_C$).

**Scale** The x-scales of the histograms from the side views can be set to one of two modes. The scales can either be fitted to the minimum and maximum value of each individual histogram (see Fig. 5) or to the minimum and maximum value across all histograms from the same column (see Fig. 4). In the first case, it is easier to compare the information within a single histogram, and in the second case, it is easier to compare the information from different data entities to investigate, e.g., which phases took longer overall ($G_C$).

**Zoom** The users can zoom in and out of the time-dependent main views. The zooming is synchronized between the views ($G_S$). This is particularly useful for high-frequent data like device measurements (see Fig. 7).

**Highlight** Highlighting allows users to select subsets of the procedures to compare them against the remaining procedures. To do so, the users can draw yellow highlight constraint boxes in any of the reference, main, or side views (e.g., see Fig. 5). Subsequently, the visual representations of all procedures whose values lie in the given box are kept colored while the visual representations of those which do not fulfill the constraint are colored gray. This highlighting is applied synchronously across all views ($G_S$). It allows comparing subgroups of procedures based on their values ($G_C$), providing a deeper insight into the workflows the different procedures followed ($G_W$).

**Filter** To compare specific subsets of procedures or even one procedure against another ($G_C$), users have the option to filter out procedures based on their values. Once a highlighted subset was selected, the users can either remove all other non-highlighted procedures or remove the highlighted procedures themselves to drill deeper into the data. Filtering can also be used to remove outliers that otherwise skew aggregated information.

**Auxiliary** In addition to the interaction techniques above, there are minor techniques to support the analysis. The layout of the application can be customized by extending, shrinking, or hiding the views of entire data categories or individual entities to compare information of interest more easily ($G_C$). Additionally, the investigation of the procedure reference view is supported by allowing users to reorder the procedures according to the depicted values, making correlations easier to detect ($G_C$).

## Evaluation and results

We performed an evaluation with 12 participants (9 with medical and 3 with technical background). They were either medical students (7/12), had a Master’s degree (3/12), or a medical license (2/12). All participants had between 0 and 7 years of professional experience with a mean of 2 years. One of them was the medical doctor with whom we had developed the application, the other participants had never seen it before.

The evaluation took one hour. For the first ten minutes, the participants were shown an explanatory video introducing the application. Afterward, they were asked to answer a form that consisted of three parts: Tasks, Hypotheses and Open Feedback, and Usability. The video and the form are attached in the supplementary information.

### Tasks

**Fig. 8:**
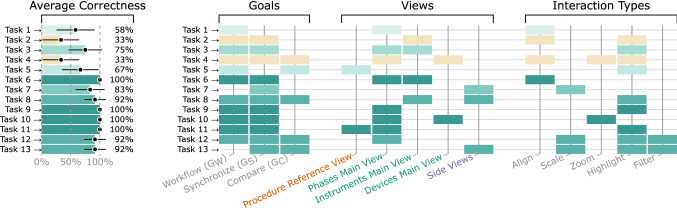
The figure shows for each task how well the participants could solve it (left) and which aspects it covered (right). Each row corresponds to a single task. In each row, on the left, the average correctness is given, and the corresponding 95%-confidence interval. On the right, there is a rectangle in a row for each aspect covered by the corresponding task. The fill color of each rectangle in a row is the same, representing the average correctness

The participants were given 13 tasks (summarized in Table 1) to solve using the application. Fig. 8 (right) shows an abstraction of which aspects were covered by which tasks. (Note that even though some main goals implicitly include others (e.g., $G_W$ includes $G_C$ and $G_S$), we only state the goals in the figure which were explicitly part of each task.) Our goal with the tasks was to evaluate whether experts are able to analyze and compare synchronized, post-operative surgical data to improve their understanding of the underlying workflows. Some consecutive tasks were also designed to reflect a sequence of analysis steps building on each other that might occur in a real-world scenario.

**Table 1:** Summary of the evaluation tasks. Tasks 1-5 were performed on proctocolectomy surgeries [10], tasks 6-8 on rectal resections [10], tasks 9-10 on sigmoid resections [10], and tasks 11-13 on gallbladder resections [16]

Task	Summary
1	Align surgical procedures at beginning of specific phase and identify which preceding phases were visited in any of the procedures after the alignment point
2	Change alignment point and use highlighting to check whether two given instruments were used simultaneously in any of the procedures
3	Determine which instruments are no longer used after a certain point
4	Change alignment point, zoom in, and use highlighting to inspect during which phase a peak in the measurements of a specific device occurred
5	Compare the overall duration of the highlighted procedures from the previous task against the remaining ones
6	Change alignment point and determine which instrument was used most after it
7	Use summary histograms to determine how many procedures had unusually high average values for a certain device
8	Highlight corresponding procedures from previous task and determine which instrument is used the longest at a stretch in them
9	Highlight the procedures which entered the NOOP phase and identify the phase during which it occurred (see Fig. 7)
10	Zoom in on the interval of the NOOP phase to find that a certain device measurement suddenly increased during the interval (see Fig. 7)
11	Highlight the procedures that took longer overall and inspect how much of their duration was spent in a certain phase
12	Filter for the longer procedures to find that they generally take particularly long w.r.t. exactly one of two specific phases, however, never w.r.t. both
13	Filter and highlight to compare the longest two procedures regarding for which instrument their usage time differed the most

**Results.** The *average correctness* for each task, i.e., the percentage of participants who could solve it correctly, is depicted in Fig.8 (left). The overall average correctness was 78.8%. While the participants struggled with tasks 2 and 4, they could solve almost all tasks correctly later on.

### Hypotheses and open feedback

We asked the participants whether they could verify existing hypotheses or come up with new ones using the application. In addition, we asked them for potential improvements of the application.

**Results** The open feedback showed that the participants could generate new hypotheses using the application. To give an example for a possible hypothesis: *For a certain set of procedures, a specific phase was particularly difficult, indicated by a higher occurrence of the exceptional NOOP phase during that phase*. A participant followed this investigation, mentioning in the free feedback that, for a selected subset, they found that “[p]rocedure[s] that experience diff[i]culties often only do so in one phase.” Another hypothesis proposed and validated by a participant was that during the “NOOP [phase], the gas pressure changes” for the sigmoid resection surgeries [10]. In addition, the participants found correlations between the duration of different phases as well as constraints as to when certain instruments are used. It turned out that the participants barely had any pre-existing hypotheses before the evaluation, so they focused on investigating those that came up during the evaluation. Regarding potential improvements, they said that the application would benefit from being more self-explanatory, e.g., by using more annotations. They also suggested some minor improvements of the interface, such as more precise descriptions of the x-axes in the summary histograms, or a simplification of certain interactions. Also, even for the larger set of 80 procedures from *Cholec80* [16], none of the participants experienced performance issues.

### Usability

We presented the participants the questions from the *System Usability Scale* (SUS) [17] to evaluate the usability of the application. The SUS relies on ten statements covering different usability aspects, each followed by a 5-point Likert scale question asking for the agreement of the participant with the statement. It yields an overall value between 0 (worst) and 100 (best).

**Results** The questionnaire yielded a score of 57.73. We give below the two statements for which our application received the best feedback on average. At that, 1 corresponds to “strongly disagree” and 5 to “strongly agree”:

- “I thought there was too much inconsistency in this system.” (Average: 1.9 $\approx$ disagree)
- “I found the various functions in this system were well integrated.” (Average: 3.8 $\approx$ agree)

The two questions for which the feedback was on average least in favor of our application are the following:

- “I felt very confident using the system.” (Average: 2.3 $\approx$ disagree-neutral)
- “I think that I would like to use this system frequently.” (Average: 2.4 $\approx$ disagree-neutral)

All other statements received feedback that was on average between the highest and the lowest rating stated above.

## Discussion and future work

The tasks summarized in Table 1 reflect a variety of questions that could be answered using our application. Accordingly, the participants were able to find and compare various patterns in the data. Together with the free feedback, this shows that the application facilitates the understanding of surgical workflows and the formation and validation of new hypotheses about the data, e.g., regarding the occurrence of the exceptional NOOP phase. Since barely any participants had hypotheses about the data sets prior to the evaluation, they could only investigate the ones that came up throughout it. In addition, it needs to be noted that some participants found the application complex to use, as a SUS score of 57.73 reflects. The main indicator was that the participants did not feel “very confident when using the system” and they did not have a strong wish “to use [it] frequently.” One reason is probably that some of the participants do not work in the area of workflow modeling and, thus, had little experience with the domain and have little use for the application in their daily work.

Another possible reason is that the tasks might have been too complex too early in the evaluation. Particularly task 4 (see Table 1), where multiple operations were necessary before assessing patterns across different views (devices and phases main view). Yet, later in the evaluation, such complex tasks could be solved successfully. For instance, task 10 also contains comparisons across views and was solved with 100% average correctness. This suggests that a training effect might be the reason for the improving performance of the participants. However, this claim should be made with caution as the tasks differ in the aspects that they cover and are, thus, not fully comparable. Such a training effect could be more visible if we had presented the tasks in random order. However, there were two reasons against that. On the one hand, we would have had to split up the twelve participants into smaller groups, reducing the sample size to a point where it would have been difficult to draw representative conclusions. On the other hand, some of the tasks depended on each other contextually, so a true randomization would not have been reasonable.

Despite these limitations, the participants found “the various functions [...] well integrated,” and, toward the end of the evaluation, were able to solve almost all tasks correctly. Therefore, we could improve on existing approaches by not only providing single views focused on specific types of data but, instead, presenting an application that allows experts to analyze synchronized post-operative information from diverse data sources.

In the future, we want to include the interface improvements suggested by the participants. Moreover, we want to investigate how the application can be made more explanatory, employing techniques from visual storytelling.

Additionally, we want to reflect on what other information might be interesting to include in the design. As mentioned, information like the surgeon skill and procedure similarity can be included in the procedure reference view, and additional derived values can be included in the side views. Meanwhile, the integration of hierarchical relations between surgical events of different granularity (like phases > steps > activities [6]) requires a more thorough revision of the design.

## Conclusion

We described a general scenario for the interactive visual exploration of surgical process data, for which we derived the underlying main analysis goals. We created a design to address these goals based on multifaceted open-source datasets containing a variety of data sources, making it applicable to other datasets with comparable characteristics. We implemented the design and evaluated it with twelve participants across four types of surgeries. The evaluation showed that after initial challenges, the participants were able to solve various analysis tasks and to come up with new hypotheses, improving their understanding of the surgical workflows. Currently, the application is an expert tool, which gives reason for future investigations into more explanatory approaches. Future work should also include investigations on how to integrate hierarchical information.

## Supplementary Information

Below is the link to the electronic supplementary material.

Supplementary file 1 (pdf 352 KB)

Supplementary file 2 (mp4 300366 KB)
