# Supplementary material for: Interactive visual exploration of surgical process data
Source: Int J Comput Assist Radiol Surg. 2022 Oct 21;18(1):127–37. doi: 10.1007/s11548-022-02758-1 (PMC9883333; doi:10.1007/s11548-022-02758-1)
Supplement: Supplementary file 1 — (pdf 352 KB) [file 11548_2022_2758_MOESM1_ESM.pdf]

Evaluation Questionnaire

Thank you very much for participating in our evaluation! We appreciate you taking your time.

The questionnaire contains the following sections:

0. Agreement to anonymous processing of data
1. Personal information
2. Concrete tasks and questions regarding proctocolectomy procedures [1]
3. Concrete tasks and questions regarding rectal resection procedures [1]
4. Concrete tasks and questions regarding sigmoid resection procedures [1]
5. Concrete tasks and questions regarding gallbladder resection procedures [2]
6. Open questions about your insights
7. Questions regarding the usability

By participating in the evaluation, you agree that your answers will be stored anonymously for scientific usage.

[1] Maier-Hein, Lena, et al. "Heidelberg colorectal data set for surgical data science in the sensor operating room." Scientific data 8.1 (2021): 1-11.
[2] Twinanda, Andru P., et al. "Endonet: a deep architecture for recognition tasks on laparoscopic videos." IEEE transactions on medical imaging 36.1 (2016): 86-97.

* Required

0. Agreement to anonymous processing of data

1. I agree that my data will be analyzed anonymously and published scientifically. *

Mark only one oval.

- ( ) Yes
- ( ) No

1. Personal information

2. (1.1) What is your background? *

Mark only one oval.

- Medical
- Technical
- Other: ______

3. (1.2) If you have a medical background, what is your highest academic degree? *

Mark only one oval.

- -- I do not have a medical background --
- General higher education entrance qualification (Allgemeine Hochschulreife)
- Medical license

4. (1.3) If you do NOT have a medical background, what is your highest academic degree? *

Mark only one oval.

- -- I have a medical background --
- General higher education entrance qualification (Allgemeine Hochschulreife)
- Bachelor's degree
- Master's degree
- Doctor's degree

5. (1.4) How many years have you spent as a medical practitioner? *

______

6. (1.5) If you do NOT have a medical background, how many years have you spent in your current profession?

______

2. Proctocolectomy procedures

Let us take a look at the proctocolectomy procedures. To do so, make sure that the surgery type "Prokto" is selected at the top in the toolbar on the left.

Then, align the surgeries at the start of the phase “Dissection and resection of rectum”.

7. (2.1) After entering a phase that late in the surgery, it gets less likely that the surgeon has to go back to one of the early phases. Still, there might be the need to so. Based on the data, which of the previous phases did a surgeon have to step BACK into after entering the phase “Dissection and resection of rectum”, i.e., which previous phases show up again after the alignment point? (Multiple answers possible) *

Check all that apply.

- [ ] --none--
- [ ] Preparation and orientation at abdomen
- [ ] Dissection of lymphnodes and blood vessels
- [ ] Retroperitoneal preparation to lower pancreatic border
- [ ] Retroperitoneal preparation of duodenum and pancreatic head
- [ ] Mobilizing the sigmoid and the descending colon
- [ ] Mobilizing the spenic flexure
- [ ] Mobilizing the tranverse colon
- [ ] Mobilizing the ascending colon

Now, align the surgeries at the start of phase “Preparing the anastomosis extraabdominally” which comes right after the phase above. Let us take a closer look at the usage of the different instruments after the start of this phase. For instance, the instruments "Grasper" and "Ligasure" are both used throughout the 30 minutes after the alignment point. However, it is not yet clear if they are also used simultaneously. To investigate this, highlight the procedures that used the "Grasper" during the 30 minutes after the current alignment point.

8. (2.2) Does any of the highlighted procedures also use the instrument "Ligasure" in the 30 minutes after the alignment point? *

Mark only one oval.

- ◯ Yes, there is at least one procedure that uses both instruments in the given interval.
- ◯ No, there is no procedure that uses both instruments in the given interval.

Let us now investigate whether there are any instruments that will not be necessary anymore after stepping into the phase “Preparing the anastomosis extraabdominally”. To make it easier, you may reset the highlights.

9. (2.3) Which instruments are NO LONGER used after the start of the phase “Preparing the anastomosis extraabdominally”? (Multiple answers possible)

Check all that apply.

- [ ] --none--
- [ ] Aspirator
- [ ] Clipper
- [ ] Grasper
- [ ] Harmonic Scalpel
- [ ] I-Hook
- [ ] J-Hook
- [ ] Ligasure
- [ ] Needle Holder
- [ ] Scissors
- [ ] Silicone Drain
- [ ] Stapler
- [ ] Swap Holder

Now, let us focus on the device data. To do so, set the alignment point back to the start of the first phase "Preparation and orientation at abdomen". Then, scroll down to the "Devices" view. For the time being, you may collapse the "Instruments" view by clicking on the button next to the heading.

The curves of some devices show sudden peaks. Consider, e.g., the curve for “Current gas pressure”. Between approximately 01:29:00 and 01:32:00, the measured values appear to jump to the maximum value of 255 for some surgeries. To get a closer look at the behavior, zoom into the region. For better readability, you may click on "Click to expand" next to the label of “Current gas pressure” to expand the corresponding row. Then, highlight the procedures that have a high value for “Current gas pressure” during that interval. (To highlight the procedures with high values, the corresponding highlight brush needs to start in the upper half of the chart and reach up to its top edge.)

10. (2.4) Using the "Phases" view, in which phase(s) are the corresponding highlighted procedure(s) during the interval between 01:29:00 and 01:32:00? (Multiple answers possible) *

Check all that apply.

- [ ] Preparation and orientation at abdomen
- [ ] Dissection of lymphnodes and blood vessels
- [ ] Retroperitoneal preparation to lower pancreatic border
- [ ] Retroperitoneal preparation of duodenum and pancreatic head
- [ ] Mobilizing the sigmoid and the descending colon
- [ ] Mobilizing the spenic flexure
- [ ] Mobilizing the tranverse colon
- [ ] Mobilizing the ascending colon
- [ ] Dissection and resection of rectum
- [ ] Preparing the anastomosis extraabdominally
- [ ] Preparing the anastomosis intraabdominally
- [ ] Placing stoma
- [ ] Finishing the operation
- [ ] NOOP

11. (2.5) Using the "Procedures" view, how does the duration of the highlighted procedure(s) differ from the duration of the other procedures? *

Mark only one oval.

- ( ) The highlighted procedure(s) tend to be shorter.
- ( ) The highlighted procedure(s) tend to be longer.
- ( ) The highlighted procedure(s) have a mixed duration, both shorter and longer.

3. Rectal resection procedures

Let us now look at the rectal resection procedures. To do so, select the surgery type "Rektum".

We want to investigate which instruments are used the most towards the end of this surgery type. To do so, align the surgeries at the start of the phase “Preparing the anastomosis extraabdominally”. Then, focus on the "Instruments" view.

12. (3.1) Which instrument is used the most after the start of the phase “Preparing the anastomosis extraabdominally” until the end of the procedure?

Mark only one oval.

- Aspirator
- Clipper
- Grasper
- Harmonic Scalpel
- I-Hook
- J-Hook
- Ligasure
- Needle Holder
- Scissors
- Silicone Drain
- Stapler
- Swap Holder

We now want to make use of the summary histograms on the right. To do so, make sure that the "Scale for summary histograms" in the toolbar on the left is set to "Individual". Then, scroll down to the "Devices" view. Here, focus on the row for the device "Current gas pressure". In that row, inspect the corresponding summary histogram on the right depicting the "Average value across surgery". On the right end of the histogram, there is an outlier bar representing procedures of an average value of around 75.73.

13. (3.2) How many procedures have an average "Current gas pressure" value of around 75.73, i.e., are contained in the rightmost bar of the "Current gas pressure" summary histogram? *

Mark only one oval.

- 0
- 1
- 2
- 3
- 4
- 5
- 6
- 7
- 8

14. (3.3) Highlight the corresponding bar and then refer to the “Instruments” view. Which instrument is used the longest at a stretch by the highlighted procedure(s)? (Welches Instrument wird von den hervorgehobenen Eingriffen am längsten am Stück verwendet?)

Mark only one oval.

- Aspirator
- Clipper
- Grasper
- Harmonic Scalpel
- I-Hook
- J-Hook
- Ligasure
- Needle Holder
- Scissors
- Silicone Drain
- Stapler
- Swap Holder

4. Sigmoid resection procedures

Let us now look at the sigmoid resection procedures. To do so, set the surgery type to "Sigma".

At the bottom of the "Phases" view, you can see a phase called "NOOP". This phase is entered if something exceptional occurs during a procedure. Highlight the procedures which entered this phase.

15. (4.1) Telling by the "Phases" view, during which phase of the highlighted procedure(s) did the exceptional "NOOP" phase occur? *

Mark only one oval.

- Preparation and orientation at abdomen
- Dissection of lymphnodes and blood vessels
- Retroperitoneal preparation to lower pancreatic border
- Retroperitoneal preparation of duodenum and pancreatic head
- Mobilizing the sigmoid and the descending colon
- Mobilizing the spenic flexure
- Mobilizing the tranverse colon
- Mobilizing the ascending colon
- Dissection and resection of rectum
- Preparing the anastomosis extraabdominally
- Preparing the anastomosis intraabdominally
- Placing stoma
- Finishing the operation

We now want to inspect the development of the device values during the "NOOP" phase, and whether there was any unusual behavior. To do so, first use the temporal brush to zoom in closely on the interval where the "NOOP" phase occurred in the "Phases" view. Then, scroll down to the "Devices" view. For this example, let us focus on the values of the "Current gas flow rate".

16. (4.2) During the second half of the "NOOP" phase, how does the "Current gas flow rate" develop? (Beware that there are two devices that start with "Current gas..." to not mistake them with each other.)

Mark only one oval.

- It suddenly increases.
- It suddenly decreases.
- It stays approximately the same.

5. Gallbladder resection procedures

We now come to the last of the surgery types, the gallbladder resection procedures. Accordingly, select the surgery type "Cholec".

To get a better understanding of why certain procedures take longer than others, we want to compare the procedures with a longer duration against those with a shorter duration. To do so, go to the "Procedures" view and roughly highlight the half of the procedures which have a longer duration.

When looking at the "Phases" view, you will find that there are two phases which took by far the longest. These phases are the "Calot Triangle Dissection" and the "Gallbladder Dissection". To answer the next question, make use of the summary histograms on the right regarding the "Time spent in phase [min]".

17. (5.1) How long did the longest procedures spend in the phase "Calot Triangle Dissection"? *

Mark only one oval.

- Between 60 and 70 minutes
- Between 70 and 80 minutes
- Between 80 and 90 minutes

Let us now focus exclusively on the procedures which took longer. To do so, click on "Remove Non-Highlighted Procedures" in the toolbar on the left. We now want to inspect whether there are procedures which take extraordinarily long with respect to both of the two phases stated above, or whether procedures generally only take extraordinarily long with respect to at most one of those two phases.

To do so, set the "Scale for summary histograms" in the toolbar on the left to "Individual". Then, refer to the summary histograms on the right regarding "Time spent in phase [min]". Focus on the histogram corresponding to the phase "Calot Triangle Dissection". In this histogram, highlight all procedures taking extraordinarily long, i.e., highlight the two rightmost bars in the histogram. Then, focus on the histogram corresponding to the phase "Gallbladder Dissection" which depicts the "Time spent in phase [min]".

18. (5.2) Do any of the highlighted procedures also occur in the two rightmost bars of the "Time spent in phase [min]"-histogram corresponding to the phase "Gallbladder Dissection"? *

Mark only one oval.

- Yes
- No

We now want to compare the two procedures which took the longest. To do so, highlight them in the "Procedures" view (if the corresponding bars are too small on your screen to highlight, you may refer to * below). Then, remove all other procedures from the view by clicking "Remove Non-Highlighted Procedures" in the toolbar on the left. With only the two longest procedures remaining, highlight one of them to be able to compare it to the other. We want to compare the procedures with respect to their instrument usage.

(*: If the bars of the two longest procedures are too small on your screen to highlight them meaningfully, it may help to zoom into your browser window by pressing the CTRL and + key simultaneously (or Command and + under Safari).)

19. (5.3) For which of the instruments is the difference between the two procedures the largest regarding the total instrument usage time? (Hint: It becomes easier if the "Scale for summary histograms" in the toolbar on the left is set to "Shared".) *

Mark only one oval.

- Grasper
- Bipolar
- Hook
- Scissors
- Clipper
- Irrigator
- SpecimenBag

6. Insights

Do you have an own assumption about the data that you would like to verify? If yes, you can do so, now.

20. (6.1) Did you have any existing assumptions about the data that you could verify or falsify in the previous usage? If yes, please describe them briefly. *

21. (6.2) Did you come up with new assumptions regarding the data throughout the investigations? If yes, please describe them briefly. *

22. (6.3) Is there any functionality that you were missing which would have improved your investigations? *

7. Usability

In the following, you will be asked 10 questions concerning the usability of the system.

The possible replies are based on a 5-point Likert scale, ranging from "Strongly Disagree" to "Strongly Agree":

1	2	3	4	5
Strongly Disagree	Disagree	Neutral	Agree	Strongly Agree

23. (7.1) I think that I would like to use this system frequently. *

Mark only one oval.

	1	2	3	4	5	
Strongly Disagree	◯	◯	◯	◯	◯	Strongly Agree

24. (7.2) I found the system unnecessarily complex. *

Mark only one oval.

	1	2	3	4	5	
Strongly Disagree	◯	◯	◯	◯	◯	Strongly Agree

25. (7.3) I thought the system was easy to use. *

Mark only one oval.

	1	2	3	4	5	
Strongly Disagree	◯	◯	◯	◯	◯	Strongly Agree

26. (7.4) I think that I would need the support of a technical person to be able to use this system. *

Mark only one oval.

	1	2	3	4	5	
Strongly Disagree	◯	◯	◯	◯	◯	Strongly Agree

27. (7.5) I found the various functions in this system were well integrated. *

Mark only one oval.

	1	2	3	4	5	
Strongly Disagree	◯	◯	◯	◯	◯	Strongly Agree

28. (7.6) I thought there was too much inconsistency in this system. *

Mark only one oval.

	1	2	3	4	5	
Strongly Disagree	⬭	⬭	⬭	⬭	⬭	Strongly Agree

29. (7.7) I would imagine that most people would learn to use this system very quickly. *

Mark only one oval.

	1	2	3	4	5	
Strongly Disagree	⬭	⬭	⬭	⬭	⬭	Strongly Agree

30. (7.8) I found the system very cumbersome (umständlich) to use. *

Mark only one oval.

	1	2	3	4	5	
Strongly Disagree	⬭	⬭	⬭	⬭	⬭	Strongly Agree

31. (7.9) I felt very confident using the system. *

Mark only one oval.

	1	2	3	4	5	
Strongly Disagree	⬭	⬭	⬭	⬭	⬭	Strongly Agree

32. (7.10) I needed to learn a lot of things before I could get going with this system. *

Mark only one oval.

	1	2	3	4	5	
Strongly Disagree	◯	◯	◯	◯	◯	Strongly Agree

Done!

Thank you very much for participating in the evaluation!

This content is neither created nor endorsed by Google.

Google Forms
